# Alveolar Lipid–Macrophage Networks at the Intersection of Pulmonary Fibrosis

**DOI:** 10.3390/cells15080668

**Published:** 2026-04-09

**Authors:** Simon H. Apte, Viviana P. Lutzky, Penny L. Groves, Daniel C. Chambers

**Affiliations:** 1 Queensland Lung Transplant Service, The Prince Charles Hospital, Chermside, QLD 4032, Australia; 2 Faculty of Health, Medicine and Behavioural Sciences, The University of Queensland, St. Lucia, QLD 4072, Australia; 3 Australian Centre for Cellular Aging (ACCA), The Prince Charles Hospital, Chermside, QLD 4032, Australia

**Keywords:** idiopathic pulmonary fibrosis, alveolar macrophages, lipid metabolism, surfactant homeostasis, macrophage heterogeneity, cholesterol handling, lysophospholipid signalling, eicosanoids, fibrosis, immunometabolism

## Abstract

**Highlights:**

**What are the main findings?**

- Alveolar macrophages are specialised for lipid handling in the surfactant-rich alveolus, and macrophage heterogeneity in idiopathic pulmonary fibrosis reflects variation within this core lipid-handling programme.
- Profibrotic macrophage states in IPF are associated with altered lipid processing and signalling, including sterol handling, lysophospholipid pathways, and eicosanoid balance, and these programmes remain pharmacologically modifiable in humans.

**What are the implications of the main findings?**

- A lipid-metabolic framework provides a coherent basis for comparing macrophage populations across IPF datasets, experimental models, and disease stages.
- Interventions that preserve or restore macrophage lipid-handling capacity may be more effective than strategies targeting individual downstream inflammatory or fibrotic mediators.

**Abstract:**

Idiopathic pulmonary fibrosis (IPF) is characterised by progressive parenchymal remodelling, driven by epithelial dysfunction, fibroblast activation, and altered immune regulation within the distal lung. Alveolar macrophages (AMs) reside in a surfactant-rich environment and are specialised for continuous lipid handling, yet the significance of this metabolic role for macrophage heterogeneity and fibrotic progression has remained incompletely integrated across studies. In this review, we synthesise evidence from human lung tissue, experimental models, lipidomic analyses, and clinical investigations to place macrophage populations described in IPF—including FABP4-high homeostatic cells and SPP1-associated disease-enriched states—within a unified lipid-metabolic context. We show that macrophage heterogeneity in IPF can be understood as a variation within a core lipid-handling programme rather than the emergence of distinct macrophage lineages. Profibrotic macrophage states are characterised by altered lipid processing and signalling, including dysregulated sterol handling, lysophospholipid pathways, and eicosanoid balance, which impair surfactant turnover and contribute to fibroblast activation. Importantly, experimental and clinical data indicate that macrophage lipid-metabolic programmes remain modifiable, although definitive disease-modifying efficacy in IPF has yet to be established. Framing macrophage states within a lipid-metabolic framework provides a coherent basis for interpreting heterogeneous datasets and supports the rationale for therapeutic strategies aimed at stabilising or restoring macrophage lipid handling in fibrotic lung disease.

## 1. Introduction: Lipid Homeostasis at the Alveolar Interface

Idiopathic pulmonary fibrosis (IPF) has traditionally been framed as a disorder of aberrant wound healing, driven by recurrent epithelial injury. Increasing evidence supports a broader interpretative framework in which disturbances in alveolar lipid homeostasis emerge as a prominent and recurrent feature of disease. In human studies and experimental models, disrupted surfactant biochemistry, impaired epithelial lipid synthesis, and macrophage metabolic dysfunction are frequently observed alongside epithelial injury and fibrotic remodelling, suggesting that lipid disequilibrium is tightly interwoven with core pathogenic processes in IPF.

The alveolus represents a uniquely lipid-rich and low-glucose niche (reviewed in [1]). Its luminal surface is coated with pulmonary surfactant, a complex mixture dominated by phospholipids (particularly phosphatidylcholine (PC) species such as dipalmitoyl-phosphatidylcholine (DPPC)) together with neutral lipids, principally cholesterol, and specialised surfactant proteins (reviewed in [2]). This lipid layer is essential for lowering surface tension, preventing alveolar collapse at end-expiration, and providing a first line of defence against inhaled pathogens (reviewed in [3]).

Maintenance of this lipid environment depends on tight metabolic coupling between alveolar type II (AT2) epithelial cells and alveolar macrophages (AMs). AT2 cells synthesise and secrete surfactant lipids through high-flux PC and neutral-lipid pathways, reflecting exceptional lipid-metabolic capacity. AMs reside within the surfactant layer and perform much of the downstream turnover, forming a coordinated epithelial–macrophage lipid circuit (reviewed in [4]). AM identity is imprinted by granulocyte–macrophage colony-stimulating factor (GM-CSF), which drives a PPARG-centred transcriptional programme required for lipid handling, surfactant catabolism, and long-term AM maintenance [5,6].

In healthy lungs, this circuit supports a FABP4^+^, lipid-catabolic macrophage identity characterised by high expression of PPARG, FABP4, ABCA1, ABCG1, and scavenger receptors, and dominating the AM compartment in health [7,8,9,10].

This review focuses on lipid–macrophage circuits in the alveolus, arguing that disruption of surfactant–macrophage lipid homeostasis provides a unifying framework linking epithelial injury, macrophage metabolic state, and progressive fibrotic remodelling. We first define the healthy alveolar lipid landscape, then detail the lipid-metabolic programmes that organise AM states, before dissecting how lipid dysregulation drives pathogenic circuits in IPF. We conclude by considering therapeutic strategies aimed at restoring lipid–macrophage homeostasis and re-establishing antifibrotic lipid tone.

## 2. The Healthy State Alveolar Lipid Landscape

### 2.1. Phosphatidylcholine and Surfactant Biophysics

The alveolar surface is coated by a lipid-rich surfactant layer dominated by phospholipids that maintain biophysical stability and immune readiness [11,12]. PC species constitute the bulk of surfactant lipids, with DPPC providing the primary surface-active component.

AT2 cells synthesise PC through de novo fatty-acid incorporation and Lands-cycle remodelling, in which lysophosphatidylcholine is reacylated by lysophosphatidylcholine acyltransferase-1 (LPCAT1) to generate saturated PC species essential for monolayer stability under respiratory compression [13,14]. LPCAT1 is highly enriched in AT2 cells, where its activity determines DPPC abundance [15]. Loss of LPCAT1 alters surfactant composition, associates with impaired AT2 progenitor renewal, and increases susceptibility to fibrotic injury [16,17].

Surfactant phospholipids incorporate saturated and unsaturated fatty acids, with chain length and saturation influencing surface-tension behaviour and susceptibility to oxidation [12,18].

### 2.2. Cholesterol Homeostasis and Sterol Trafficking

Cholesterol constitutes a smaller but functionally critical fraction of surfactant lipids, modulating membrane fluidity and resistance to collapse. Excessive cholesterol disrupts surfactant homeostasis and induces AT2 stress, whereas insufficient cholesterol impairs film stability (reviewed in [2,19,20]).

Following surfactant uptake, AMs rely on ATP-binding cassette (ABC) transporters to export excess cholesterol and maintain sterol homeostasis. In macrophages, this function is divided between two non-redundant transporters: ABCG1, which supports cholesterol efflux to lipid-rich acceptors within the surfactant milieu, and ABCA1, which primarily transfers cholesterol to lipid-poor apolipoproteins such as apolipoprotein A-I. In the alveolus, GM-CSF signalling is critical for sustaining a PPARG-dependent programme that preferentially supports ABCG1-mediated efflux. In GM-CSF deficiency, ABCG1 expression is markedly reduced; although ABCA1 may be upregulated, this shift in efflux machinery is insufficient to prevent intracellular sterol accumulation [21]. Consistent with this functional hierarchy, genetic deletion of ABCG1 alone impairs cholesterol export, resulting in sterol-laden, foamy AMs, progressive pulmonary lipidosis with abnormal AT2 lamellar body structure, and exaggerated inflammatory injury, establishing ABCG1 as a dominant regulator of alveolar lipid balance and lung homeostasis [22,23].

Oxysterols (oxygenated forms of cholesterol) generated through enzymatic and oxidative pathways exert immunomodulatory effects but are tightly constrained in health via liver X receptor (LXR)-dependent feedback [24,25].

### 2.3. Fatty-Acid Metabolism in the Alveolus

Pulmonary surfactant homeostasis imposes a uniquely high demand for fatty-acid metabolism in the alveolus. AT2 epithelial cells serve as the primary synthetic compartment, generating surfactant phospholipids de novo from circulating lipid substrates and intracellular fatty-acid synthesis pathways, and packaging these lipids into lamellar bodies for regulated secretion into the alveolar space. This process requires continuous fatty-acid supply and phospholipid remodelling to preserve surfactant composition and biophysical function (reviewed in [26]).

Following secretion, a substantial fraction of surfactant lipids is retrieved from the airspace and processed by AMs, which constitute a major catabolic and clearance compartment for surfactant-derived fatty acids and sterols. Efficient handling of this lipid burden is essential for alveolar homeostasis: genetic or signalling-mediated disruption of macrophage lipid-processing pathways leads to intracellular lipid accumulation, foam-cell formation, pulmonary lipidosis, and inflammatory lung injury [21,22,23]. Together, AT2 cells and AMs form a metabolically integrated system in which fatty acids are continuously synthesised, remodelled, recycled, or degraded to maintain surfactant balance and prevent lipid overload.

Consistent with this metabolic specialisation, single-cell RNA-sequencing studies of healthy human lung consistently identify a FABP4^+^ AM population, defined by expression of fatty-acid binding protein 4 (FABP4), a cytosolic lipid chaperone that facilitates intracellular fatty-acid trafficking and lipid handling. This transcriptional programme corresponds to the canonical homeostatic AM state and is progressively lost in inflammatory and fibrotic lung disease, underscoring fatty-acid metabolic competence as a defining property of macrophage homeostasis in the distal lung [8,9,10,27].

### 2.4. Eicosanoid Balance in the Healthy Alveolus

Liberation of arachidonic acid from membrane PC provides a shared substrate for multiple eicosanoid pathways in the alveolus. In the healthy lung, however, eicosanoid production is selectively biased rather than evenly distributed. Prostaglandin E_2_ (PGE_2_), a bioactive lipid mediator generated via cyclooxygenase pathways, represents the dominant eicosanoid signal under basal conditions. PGE_2_ acts on lung fibroblasts through EP2 and EP4, the principal PGE_2_ receptors coupled to cyclic AMP (cAMP) signalling, thereby restraining fibroblast proliferation, inhibiting fibroblast–myofibroblast differentiation, and limiting collagen synthesis [28,29].

Other arachidonic acid-derived eicosanoid pathways remain biochemically available but relatively quiescent in health, preserving the capacity for rapid inflammatory or reparative responses without constitutive activation. The balance between dominant PGE_2_-mediated inhibitory signalling and these latent counter-regulatory pathways contributes to alveolar immune quiescence and tissue homeostasis [30].

### 2.5. Sphingolipids and Lysophospholipids in the Healthy Alveolus

Beyond bulk structural phospholipids, the alveolus contains low-abundance sphingolipid and lysophospholipid species that function primarily as signalling mediators rather than membrane components. Among these, sphingosine-1-phosphate (S1P) and lysophosphatidic acid (LPA) act through high-affinity receptor systems and can influence vascular and cellular behaviour at low concentrations [31,32,33]. This signalling capacity necessitates tight quantitative control under physiological conditions.

In the healthy alveolus, S1P contributes to baseline endothelial stability and barrier integrity. Primary studies in lung and vascular endothelial cells demonstrate that S1P enhances endothelial barrier function through receptor-dependent cytoskeletal reorganisation and reinforcement of intercellular junctions, including cortical actin assembly and stabilisation of adherens junctions [31,32]. These effects occur at physiologically relevant concentrations and in the absence of inflammatory stimuli, supporting a role for S1P in maintaining endothelial homeostasis within the alveolar microenvironment, despite much of the mechanistic insight deriving from vascular and developmental systems.

LPA is similarly characterised by potent signalling activity that is subject to quantitative regulation. LPA is generated extracellularly through the lysophospholipase D activity of autotaxin, which converts lysophosphatidylcholine into bioactive LPA [34]. Experimental loss-of-function studies demonstrate that autotaxin activity determines basal circulating LPA levels in vivo, with partial reduction of autotaxin leading to proportional decreases in LPA that remain compatible with normal vascular stability, whereas complete loss of autotaxin disrupts vascular development [33]. These findings indicate that LPA signalling in health is regulated around a defined physiological set-point.

Within this regulated signalling environment, resident AMs are metabolically tuned to the lipid-rich alveolus. Single-cell transcriptomic studies consistently identify FABP4^+^ homeostatic AMs as the dominant resident macrophage population in healthy human lung and demonstrate detectable baseline expression of S1P and LPA receptor genes within these cells despite such receptors not being cluster-defining markers [8,9,27]. This expression pattern is consistent with a capacity for FABP4^+^ AMs to sense sphingolipid and lysophospholipid cues as part of normal alveolar homeostasis, without implying pathway activation or pathological signalling.

Taken together, sphingolipid and lysophospholipid signalling pathways operate in the healthy alveolus as tightly regulated systems, supporting endothelial stability and immune surveillance while remaining constrained within a narrow physiological range.

## 3. Lipid-Metabolic Programmes of AMs

### 3.1. Organising Principles of Lipid-Metabolic Programming in AMs

AM states in the human lung are organised by conserved metabolic programmes that reflect adaptation to the lipid-rich alveolar environment. These states do not represent fixed lineages but instead correspond to relatively stable transcriptional configurations shaped by epithelial-derived signals, surfactant lipid flux, and cumulative metabolic stress. Experimental and human single-cell studies, including our own, demonstrate substantial interconversion between these states, establishing AM lipid programmes as plastic and environmentally conditioned rather than developmentally fixed [8,9,10,27].

To define macrophage subsets within a lipid-metabolic framework across datasets, we aligned macrophage gene sets from Groves et al. (their Supplementary Table S1) [10] to myeloid populations defined by Wendisch (their Supplementary dataset MMC2) [27]. Groves’ state-specific gene lists (e.g., FABP4-high and SPP1-associated macrophages) were filtered to retain genes with average log2 expression >0 and compared against Wendisch subset marker genes filtered at FDR < 0.05. Overlap between gene sets was quantified by intersection counts to assess correspondence between states (Microsoft Excel V16.107). This analysis identified FABP4-high macrophages as aligning with AM2 macrophages and SPP1-associated macrophages with CD163.LGMN populations, with minimal cross-alignment between non-corresponding subsets. Subsequent analyses were restricted to these aligned populations.

To define high-confidence, subset-discriminatory genes, Wendisch gene sets were further filtered using FDR < 0.05 and area under the receiver operating characteristic curve (AUC) > 0.7 relative to the opposing subset (AM2 vs. CD163.LGMN, and vice versa). These refined gene sets were subjected to functional curation using STRING (v11) [35], with genes associated with lipid handling and metabolic processes identified based on functional annotation.

Functional enrichment analysis was performed using Gene Ontology Biological Process (GO:BP) via g:Profiler [36] and KEGG pathway analyses via Enrichr [37]. Enriched terms were grouped into higher-order functional modules based on shared ontology structure and gene membership, with KEGG pathways providing independent confirmation. Modules were retained where consistent support was observed across both analyses, without inference of pathway directionality or causality. All analyses were performed using publicly available gene lists without reprocessing of the original datasets.

At the level of these curated modules, AM2-derived programmes align with FABP4-high macrophage states, whereas CD163.LGMN-derived programmes align with SPP1-associated states described across human lung datasets. These are referred to throughout this section as FABP4-like and SPP1-like states, respectively, as a matter of functional convenience and cross-study alignment, without implying fixed or lineage-restricted macrophage identities, and in keeping with a continuum model of lipid-metabolic state organisation. These lipid-metabolic programmes were supported by Gene Ontology Biological Process enrichment with independent KEGG pathway confirmation, demonstrating that each module represents a coherent and reproducible functional unit rather than an arbitrary gene grouping (Table 1).

AM identity is established early in life and maintained through sustained exposure to epithelial-derived GM-CSF and the lipid-rich alveolar niche. Central to this process is GM-CSF-dependent induction of PPARG-regulated transcriptional programmes that coordinate macrophage lipid handling with the physiological demands of continuous surfactant uptake and turnover [5,6,38].

Rather than defining a discrete macrophage subset, GM-CSF–PPARG signalling establishes a foundational metabolic configuration that is characterised by the coordinated expression of lipid-trafficking, storage, and efflux pathways. This programme supports sustained lipid accommodation in a glucose-poor environment while limiting inflammatory activation. Perturbation of this configuration—through altered epithelial lipid supply, sterol accumulation, oxidative stress, or persistent inflammatory signalling—is associated with the destabilisation of resident AM identity and the emergence of alternative macrophage programmes observed in fibrotic lung disease [9,10,27,38].

### 3.2. FABP4-like Lipid-Handling AMs

Within the GM-CSF–PPARG-dependent framework, FABP4-like AMs represent a lipid-adapted macrophage programme characteristic of the healthy human lung. At the level of coordinated transcriptional organisation, this state is defined by a set of lipid-metabolic functional modules that support intracellular lipid buffering and accommodation of continuous surfactant-derived lipid flux (Table 2).

Module-level analysis identifies recurrent programmes encompassing lipid droplet-associated lipid handling, glycerolipid metabolism and turnover, phospholipid metabolic processes, unsaturated fatty-acid biosynthesis, eicosanoid and prostanoid metabolic capacity, and membrane lipid transport (Table 2). Representative genes include *FABP4*, *PLIN2*, *PNPLA2*, *SCD*, *ACOT7*, *ALOX5*, *and ABCG1*, which recur across multiple human lung single-cell atlases [8,9,10,27,39].

Functionally, this modular configuration supports sustained intracellular lipid buffering while limiting lysosomal overload, oxidative stress, and inflammatory activation. In fibrotic lung disease, partial erosion or reconfiguration of these FABP4-aligned modules is reproducibly observed in association with expansion of inflammatory and SPP1-like macrophage states [7,9,27,40].

### 3.3. SPP1-like Macrophages and Reconfigured Lipid Processing

SPP1-like macrophages represent a disease-expanded macrophage programme consistently identified in IPF and related fibrotic lung disorders. In contrast to FABP4-like macrophages, this state is characterised not by loss of lipid-metabolic capacity, but by reorganisation of lipid-processing modules toward alternative metabolic priorities (Table 2).

Module-level analysis identifies the enrichment of programmes encompassing cholesterol transport and sterol homeostasis, endosome–lysosome lipid processing, lipoprotein particle handling, fatty-acid import and acyl-CoA metabolic processes, phospholipid metabolism, and oxidative stress-linked metabolic pathways (Table 2). Representative genes include *ABCA1*, *NPC1*, *NPC2*, *CD36*, *ACSL3*, *ACSL4*, *LAMP1*, *ASAH1*, *HMOX1*, *and CYP1B1.*

Gene Ontology enrichment of SPP1-aligned modules favours lipid-metabolic and lipid-catabolic biological processes without corresponding enrichment for lipid-binding molecular functions. This pattern distinguishes SPP1-like macrophages from FABP4-like lipid-buffering programmes and defines a reproducible macrophage configuration in which lipid-processing capacity is redeployed toward sterol stress management, lysosomal handling, and oxidative metabolism rather than intracellular lipid storage [9,10,27].

### 3.4. Inflammatory AMs

Inflammatory AM states emerge in response to epithelial injury and danger-associated signals within the alveolar space and are consistently identified across human lung single-cell atlases [8,9]. Transcriptionally, these macrophages are characterised by induction of canonical pro-inflammatory genes, including alarmins (S100A8, S100A9, S100A12), innate immune receptors and recruitment pathways (FCN1, CD14, CCR2, FPR1), and activation of NF-κB and interferon signalling networks (NFKB1, NFKBIA, TNFAIP3, STAT1, JAK1).

In our own work, this inflammatory transcriptional programme was readily induced in human AMs following exposure to lipopolysaccharide, consistent with the broader capacity of AMs to transition between lipid-metabolic configurations in response to environmental cues [10]. Across datasets, inflammatory signatures commonly appear transiently superimposed on otherwise homeostatic macrophage programmes rather than defining a stable endpoint.

From a lipid-metabolic perspective, inflammatory AMs retain much of the underlying FABP4-aligned lipid-handling architecture that is characteristic of homeostatic AMs, but exhibit modest and reversible reweighting of metabolic activity toward inflammatory signalling and effector support. Relative to AM2 macrophages, STRING-based analysis indicates subtle enrichment of the pathways supporting glycolytic entry (SLC2A3, GAPDH, PGK1), oxidative burst and redox handling (CYBB, SOD2), and ancillary lipid- or sterol-associated processes (LBR, PRELID1, VMP1, ATG3), without evidence for wholesale loss of lipid buffering, sterol efflux, or intracellular lipid storage capacity. In contrast to FABP4-associated and SPP1^hi^ macrophage states, inflammatory macrophages likely represent a more transient and less well-defined state rather than a stable, lipid-programmed population, which may account for the comparatively limited depth of current characterisation.

Together, these observations support the interpretation of inflammatory AMs as a signalling-biased configuration superimposed on a largely preserved lipid-metabolic framework. Within the broader AM continuum, inflammatory AMs therefore represent a context-dependent modulation of lipid metabolism rather than a distinct or destabilised lipid-metabolic endpoint.

### 3.5. Alveolar Macrophage Plasticity as a Lipid-Metabolic Continuum

Taken together, FABP4-like, inflammatory, and SPP1-like AM programmes are best understood as occupying positions within a continuous lipid-metabolic landscape rather than representing discrete or opposing cell identities. FABP4-like macrophages define a stable lipid-buffering configuration that is aligned with homeostatic surfactant handling, inflammatory macrophages reflect transient reweighting of this framework toward signalling and effector support, and SPP1-like macrophages represent a stress-adapted reorganisation of lipid-processing capacity under conditions of persistent epithelial dysfunction. Transitions between these states are shaped by the intensity and duration of lipid flux, inflammatory pressure, and metabolic stress within the alveolar niche, without enforcing unidirectional trajectories. This continuum model provides a coherent framework for integrating macrophage heterogeneity across datasets and underpins therapeutic strategies aimed at modulating lipid-metabolic pressures rather than eliminating specific macrophage populations.

## 4. Lipid Dysregulation and Pathogenic Circuits in IPF

### 4.1. Alveolar Epithelial Lipid Failure

IPF is characterised by a profound disruption of alveolar lipid homeostasis that is evident outside established fibrotic lesions. Transcriptomic profiling of untreated human precision-cut lung slices demonstrates coordinated suppression of lipid-metabolic programmes—including fatty-acid and acyl-CoA biosynthesis, cholesterol metabolism, and PPAR signalling—in morphologically non-fibrotic, transitional, and fibrotic regions of IPF lungs, indicating that metabolic failure is not restricted to end-stage scarred tissue [41].

While epithelial dysfunction in IPF generates multiple danger-associated signals (including accumulation of misfolded or damaged proteins that overwhelm cellular quality-control systems, as well as proteotoxic stress, oxidative injury, and telomere attrition), lipid dysregulation imposes a distinct form of pressure on AMs. Disruption of epithelial surfactant metabolism alters both the quantity and composition of lipids encountered by resident macrophages, increasing the requirement for continuous uptake, intracellular trafficking, remodelling, and disposal of structurally abnormal phospholipid and sterol species. Unlike episodic stress signals, this surfactant-derived lipid challenge is continuous and unavoidable for macrophages residing within the alveoli. In this framework, lipid dysregulation is not assumed to be an initiating insult in IPF, but rather a persistent, state-shaping pressure that stabilises macrophage identities once epithelial homeostasis is compromised.

At the epithelial level, this disruption reflects impaired PC biosynthesis and defective surfactant renewal. Pulmonary surfactant requires continuous enzymatic conversion of lysophosphatidylcholine into fully saturated PC species in order to maintain low surface tension during respiration. Lysophosphatidylcholine acyltransferase-1 (LPCAT1), which is highly expressed in alveolar type-2 (AT2) cells, catalyses this process by incorporating saturated fatty acids into PC, thereby enabling production of DPPC, the dominant surface-active lipid species in pulmonary surfactant [13,14].

In IPF and ageing lungs, reduced LPCAT1 expression together with broader lipid insufficiency compromises this phospholipid renewal pathway, leading to altered surfactant composition and impaired biophysical stability. These defects limit AT2 progenitor renewal and increase epithelial susceptibility to mechanical and oxidative injury, directly linking disrupted lipid metabolism to loss of epithelial resilience [16,17].

Consistent with these epithelial defects, human bronchoalveolar lavage studies demonstrate marked abnormalities in surfactant composition and function in IPF, including altered phospholipid class distribution, reduced proportions of disaturated PC, and impaired surface activity [11]. Detailed fatty-acid profiling further shows a shift away from palmitate-rich PC toward increased incorporation of unsaturated acyl chains, a compositional change that compromises surfactant film stability and elevates surface tension [12]. Together, these alterations produce a biophysically unstable surfactant layer enriched in unsaturated phospholipid species.

**Figure 1:**
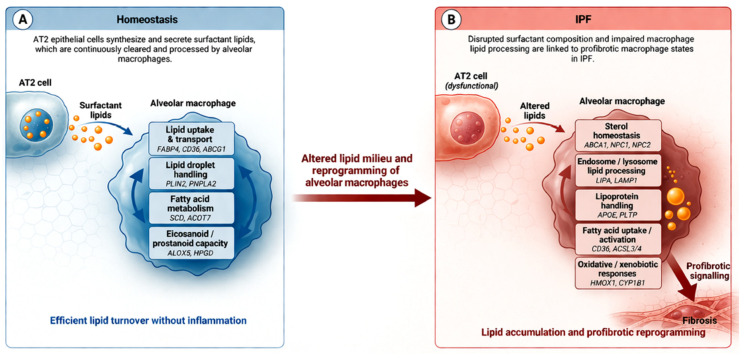
Lipid–macrophage coupling in alveolar homeostasis and its disruption in pulmonary fibrosis. (**A**) In the healthy alveolus, AT2 epithelial cells synthesise and secrete surfactant lipids that are continuously taken up and processed by alveolar macrophages through a PPARG–FABP4-centred lipid metabolic programme. This supports coordinated lipid uptake, storage, and utilisation, enabling efficient lipid turnover without inflammation. (**B**) In pulmonary fibrosis (IPF), this coupling is disrupted. Altered surfactant composition and impaired lipid processing are associated with a shift in the lipid milieu and reprogramming of alveolar macrophages toward SPP1-associated and CD163^+^ states. These macrophages exhibit altered sterol homeostasis, enhanced lysosomal lipid processing, increased fatty acid uptake, and oxidative responses, linked to lipid accumulation and profibrotic signalling. Together, these changes position alveolar macrophage heterogeneity along a lipid-handling continuum rather than discrete polarisation states.

Because surfactant turnover and clearance are largely mediated by AMs, epithelial lipid failure necessarily increases the metabolic burden placed on the resident myeloid compartment. Thus, alveolar epithelial lipid dysfunction in IPF not only undermines surfactant biophysical integrity but also establishes the upstream conditions that drive macrophage lipid stress and downstream profibrotic reprogramming (Figure 1).

Spatial transcriptomic analyses reveal that fibrotic lung tissue is structured into discrete regions reflecting progressive stages of epithelial disruption and tissue remodelling [42,43]. These datasets show that macrophage populations are regionally distributed across airspaces, with FABP4-associated and SPP1-associated states varying according to local disease severity and remodelling context [42,44]. Consistent with this, macrophages co-localise with areas of epithelial injury and fibrotic remodelling, and participate in coordinated epithelial–stromal–immune interactions within defined regions [42,43,45].

Spatial variation in stromal composition further suggests that lipid-handling capacity may differ across regions of the diseased lung. In particular, fibrotic regions are characterised by a reduction in PLIN2-positive lipofibroblast-like cells, which, in experimental systems, support alveolar type II cells through lipid provision for surfactant production and maintenance of epithelial homeostasis [46]. These findings imply that epithelial–stromal support for surfactant metabolism, and therefore local lipid availability, may be diminished in regions of active fibrosis. As macrophage lipid-metabolic programmes are closely linked to lipid uptake and processing, this provides a plausible framework through which spatial positioning could influence macrophage state. However, it is important to note that current spatial datasets are based on gene expression rather than direct measurements of lipid composition, and thus relationships between spatial location and lipid metabolism remain inferred rather than directly demonstrated (reviewed in [47]).

### 4.2. Sterol Dysregulation and the Foamy Macrophage Switch

Cholesterol metabolism is profoundly dysregulated in IPF, with sterol accumulation occurring predominantly within AMs. Lipidomic analyses demonstrate marked intracellular cholesterol enrichment in AMs, while single-cell transcriptomic studies reveal a shift away from homeostatic macrophage lipid-handling programmes toward lipid-stressed, profibrotic states, consistent with impaired sterol homeostasis and progressive lipid retention [9,38].

Failure of macrophage cholesterol efflux promotes the emergence of lipid-laden, or “foamy,” macrophage states that are characterised by lysosomal stress and inflammatory activation. Experimental studies across multiple systems show that intracellular sterol accumulation and cholesterol crystallisation trigger lysosomal destabilisation and activation of inflammasome-associated pathways, particularly NLRP3, leading to sustained cytokine production and inflammatory signalling [22,23,48]. Although these mechanistic studies were not performed in IPF per se, AMs isolated from fibrotic human lungs exhibit transcriptional programmes consistent with sterol stress, lysosomal dysfunction, and inflammasome engagement, supporting the relevance of these pathways in vivo [38].

In parallel, dysregulation of oxysterol metabolism further amplifies macrophage inflammatory tone within fibrotic lung environments. Increased expression of cholesterol 25-hydroxylase (CH25H) and accumulation of 25-hydroxycholesterol have been shown to potentiate inflammatory signalling, suppress cholesterol efflux pathways, and promote fibroblast activation and collagen synthesis in experimental models of lung injury and fibrosis [49,50,51]. These oxysterol-driven effects provide an additional layer of metabolic stress that reinforces macrophage activation under conditions of sustained lipid overload.

Together, sterol accumulation and oxysterol signalling act as powerful metabolic biasing forces that drive AMs away from lipid-buffering FABP4^+^ homeostatic states and toward SPP1^hi^ profibrotic phenotypes. Single-cell transcriptomic analyses of human IPF lungs consistently identify the expansion of SPP1^hi^ macrophage populations within fibrotic lung regions, where they display reduced expression of lipid-handling genes, increased inflammatory and lysosomal stress signatures, and robust production of osteopontin and other profibrotic mediators [7,27,40]. Through these state transitions, sterol dysregulation biases macrophages toward profibrotic signalling programmes.

### 4.3. Eicosanoid and Lysophospholipid Signalling Circuits

A defining feature of IPF is the emergence of disease-expanded macrophage populations within fibroblast-rich microenvironments, in which altered lipid metabolism reshapes the balance of bioactive lipid signalling. In this context, eicosanoids and lysophospholipids function not as isolated mediators but as components of interacting signalling pathways that link epithelial injury, macrophage lipid stress, and fibroblast activation.

In the healthy lung, prostaglandin E_2_ (PGE_2_) functions as a key antifibrotic lipid mediator, restraining fibroblast proliferation, myofibroblast differentiation, and extracellular matrix synthesis through well-characterised receptor-dependent pathways. In IPF, impaired induction of cyclooxygenase pathways and reduced PGE_2_ availability result in a loss of this inhibitory lipid tone, lowering the threshold for fibroblast activation and matrix deposition ([28,29] and reviewed in [52]). This alteration is not established as an initiating event in fibrosis, but may represent an early perturbation of the alveolar lipid environment that reduces basal restraint on fibroblast activation once epithelial homeostasis begins to fail.

Convergent experimental and clinical data support a shift toward profibrotic eicosanoid signalling in fibrotic lung disease. In experimental models of lung injury and fibrosis, leukotrienes promote inflammatory cell recruitment and fibrotic remodelling, while genetic or pharmacological disruption of leukotriene pathways confers protection from fibrosis in vivo [53]. In patients with IPF, elevated circulating metabolites of prostaglandin F_2_α are associated with disease severity and adverse outcomes, consistent with heightened profibrotic eicosanoid activity; although these observations do not resolve cellular origin or establish causality [30,54].

Within this altered lipid milieu, disease-expanded macrophage populations (particularly SPP1^hi^ states) localise to fibroblastic foci and myofibroblast-rich regions and display transcriptional programmes that are characterised by inflammatory activation, lysosomal stress, and relative loss of FABP4-associated lipid-handling identity [7,9,27,40]. Single-cell profiling studies place these macrophage states within an inflammatory effector spectrum marked by upregulation of pathways linked to arachidonic acid handling and lipid mediator biology, supporting the concept that macrophage state transitions in IPF are coupled to the rewiring of bioactive lipid signalling capacity rather than simple changes in mediator abundance (Table 2) [8,9,40].

Lysophospholipid signalling further reinforces these circuits. The autotaxin–LPA axis converts lysophosphatidylcholine into bioactive LPA, linking altered phospholipid turnover to fibroblast recruitment, survival, and matrix synthesis. While tightly constrained in the healthy lung, pathological activation of this pathway in fibrotic lung disease establishes a lipid-mediated bridge between epithelial dysfunction, macrophage activation, and mesenchymal expansion [34,55]. In human bronchoalveolar lavage, macrophage-derived autotaxin activity provides direct evidence that myeloid cells contribute to local LPA generation within fibrotic microenvironments [56].

Together, these findings support a model in which alterations in eicosanoid and lysophospholipid signalling are associated with disease-expanded macrophage states, particularly SPP1^hi^ populations. In this framework, AMs are positioned to integrate persistent lipid stress with local signalling cues and to contribute paracrine inputs that may support fibroblast activation under conditions of sustained epithelial dysfunction without implying a unidirectional or macrophage-exclusive mechanism.

### 4.4. Carbonyl Stress and AGE–RAGE Activation

The oxidative environment of the IPF lung associates with the accumulation of reactive carbonyl species generated through both glucose and lipid peroxidation, leading to the formation of advanced glycation end-products (AGEs) and related lipid-derived adducts. The principal receptor for AGEs, the receptor for advanced glycation end-products (RAGE), is highly expressed in the lung and mediates cellular responses to carbonyl stress. Human biomarker studies demonstrate an imbalance in the AGE–RAGE axis in IPF, characterised by elevated circulating AGEs and reduced levels of soluble RAGE (sRAGE), a decoy form that limits AGE signalling. An increased AGE/sRAGE ratio correlates with worse lung function, accelerated physiological decline, and reduced survival, supporting carbonyl stress as a feature of progressive parenchymal disease rather than an epiphenomenon of end-stage fibrosis [57].

In addition to systemic associations, experimental data indicate that carbonyl stress can directly influence lung macrophage behaviour. In primary human lung macrophages, exposure to advanced glycation end-products induces a RAGE-dependent inflammatory response characterised by increased chemokine and cytokine expression, reduced phagocytic capacity, and altered migratory behaviour [58]. This transcriptional and functional profile exhibits features consistent with inflammatory and SPP1^hi^ macrophage states observed in IPF lung tissue, particularly with respect to inflammatory activation, impaired clearance functions, and stress-associated remodelling programmes, supporting the view that carbonyl stress contributes to the reinforcement of maladaptive macrophage configurations within fibrotic regions of the alveolus.

Carbonyl stress, therefore, represents a persistent metabolic driver through which oxidative and lipid-derived damage signals may sustain macrophage activation over time, even in the absence of ongoing acute epithelial injury.

### 4.5. Systemic Lipid Signatures

Although the lipid dysregulation described above is centred within the alveolus, systemic lipid perturbations are detectable in the circulation of patients with IPF. Serum metabolomic profiling studies provide independent evidence of altered lipid-associated metabolic states in IPF, including alterations in lysophospholipid species [18]. In one of the few studies that directly examine circulating lipid species in IPF, Yan and colleagues identified broad changes in plasma lipid composition compared with healthy controls [59].

Multivariate analyses revealed subsets of discriminatory lipid species, predominantly PCs, phosphatidylethanolamines, sphingolipids, and triglycerides, that distinguished IPF patients from controls with high accuracy, establishing systemic lipid dysregulation as a recurrent but heterogeneous feature of the IPF phenotype [59]. However, these analyses were cross-sectional and did not assess longitudinal disease progression or survival, limiting inference regarding prognostic significance.

Importantly, circulating lipidomic and metabolomic profiles lack tissue and cell-type resolution and therefore cannot assign lipid origin to specific pulmonary compartments or immune cell populations. Nonetheless, several of the lipid classes altered in plasma, particularly PCs, lysophosphatidylcholines, and sphingolipids, are central to surfactant metabolism and AM lipid handling, rendering the distal lung a plausible contributor to the observed systemic lipid signature. Accordingly, systemic lipid changes are best interpreted as peripheral correlates of underlying pulmonary metabolic dysfunction rather than autonomous drivers of fibrotic remodelling [18,59].

Recent metabolomic studies provide preliminary evidence that circulating lipid signatures may have utility for patient stratification and treatment response assessment in IPF. Independent cohort analyses have identified reproducible alterations in serum lipid profiles, including changes in triglycerides and phosphatidylcholines, which correlate with disease severity and clinical outcomes [60,61]. In particular, longitudinal data suggest that therapy-associated shifts in lipid profiles occur only in a subset of patients and may be associated with improved outcomes, raising the possibility that circulating lipid changes may reflect treatment responsiveness in a subset of patients rather than disease activity per se.

However, these findings also highlight key limitations. Circulating lipid signals are heterogeneous across cohorts, influenced by systemic factors, and may reflect a composite of epithelial injury, metabolic adaptation, and drug effects rather than lung-specific processes. As such, while circulating lipidomics may have utility for patient stratification in clinical trials, its current role remains uncertain and requires further validation.

## 5. Therapeutic Opportunities: Reprogramming Lipid–Macrophage Circuits

Mechanistic insights from human and experimental studies indicate that macrophage dysfunction in fibrotic lung disease is not fixed but is sustained by persistent lipid stress within the alveolus. Rather than eliminating discrete myeloid subsets, emerging therapeutic strategies aim to reprogramme the macrophage metabolic state by restoring lipid handling, sterol efflux, and bioactive lipid balance. This section, therefore, focuses on lipid-centred interventions that have the potential to bias AMs away from profibrotic trajectories and toward homeostatic programmes, while acknowledging that most remain early in translation and are likely to be highly context- and stage-dependent.

### 5.1. Restoring GM-CSF–PPARG–FABP4 Programming

PPARG acts as a key transcriptional regulator of macrophage lipid-responsive gene expression, coordinating pathways involved in fatty-acid trafficking, lipid buffering, and scavenger receptor-mediated lipid uptake, while remaining dispensable for macrophage lineage commitment [62]. This positions PPARG as a regulator of macrophage metabolic configuration rather than as a primary determinant of differentiation or inflammatory fate. In the lung, epithelial-derived GM-CSF is required to licence AMs to engage PPARG-dependent lipid-handling programmes. Disruption of this axis leads to sterol accumulation, impaired surfactant processing, and heightened macrophage stress responses, whereas restoration of GM-CSF signalling reduces lipid burden and partially normalises macrophage metabolic function [38].

Pulmonary alveolar proteinosis provides strong proof-of-principle that macrophage lipid dysfunction can be therapeutically corrected in humans. In autoimmune pulmonary alveolar proteinosis, neutralising antibodies against GM-CSF impair AM lipid handling and surfactant clearance, and inhaled GM-CSF restores macrophage function, improves pulmonary gas exchange, and leads to clinical benefit [63]. In addition, pharmacological activation of PPARG has been explored in this context, with early clinical experience demonstrating the feasibility of targeting macrophage lipid metabolism in vivo using pioglitazone [64]. Although pulmonary alveolar proteinosis differs fundamentally from IPF in disease aetiology and epithelial integrity, these observations establish that macrophage lipid-metabolic programmes remain amenable to therapeutic manipulation in the human lung.

Importantly, this therapeutic logic is supported by data-driven discovery rather than candidate selection alone. In our own work, FABP4-aligned macrophage gene sets were first identified from human single-cell transcriptomic data and then interrogated using gene set enrichment approaches to infer upstream regulatory pathways and candidate perturbagens. This analysis consistently highlighted PPARG signalling and glitazones as top-ranked modulators of the FABP4-associated programme. Subsequent experimental testing confirmed that pharmacological PPARG activation reprogrammed human AMs toward a FABP4-high, lipid-handling state while preserving cellular viability and transcriptional plasticity [10]. Consistent with this, independent studies support a mechanistic link between PPARG activation and induction of lipid-handling programmes in macrophages. In murine alveolar macrophages, pharmacological activation of PPARG (e.g., rosiglitazone) induces lipid-metabolic transcriptional programmes, including the upregulation of Fabp4 and Cd36 [65], while studies in human macrophages derived from lung cancer tissue demonstrate that PPARG directly regulates FABP4 expression and that FABP4 functionally contributes to macrophage lipid-metabolic activity [66], supporting conservation of this axis across species and macrophage contexts. This closed discovery loop, linking single-cell state definition, pathway inference, and functional reprogramming in primary human cells, provides a coherent rationale for targeting macrophage lipid-metabolic plasticity in fibrotic lung disease.

Translation of GM-CSF–PPARG-centred strategies to IPF remains unproven. Unlike autoimmune pulmonary alveolar proteinosis, IPF is characterised by persistent epithelial injury, spatially heterogeneous fibrosis, and chronic inflammatory signalling, which may limit the extent to which fully homeostatic macrophage programmes can be restored. Nonetheless, these data support the continued exploration of approaches that bias the macrophage metabolic state away from lipid-stressed, profibrotic configurations and toward lipid-buffering programmes, particularly in defined disease stages or molecular contexts where macrophage plasticity is retained.

### 5.2. Enhancing Sterol Efflux and Cholesterol Handling

Failure of cholesterol efflux represents a therapeutically actionable source of lipid stress in AMs. Disruption of sterol export pathways promotes intracellular cholesterol accumulation, exaggerated inflammatory activation, and loss of lung homeostasis, positioning macrophage cholesterol handling as a potential target for intervention. In experimental systems, loss of the cholesterol transporter ABCG1 results in lipid-laden AMs, spontaneous pulmonary inflammation, and impaired lung homeostasis, directly implicating sterol efflux pathways in the maintenance of macrophage function within the alveolus [23]. These findings provide a mechanistic rationale for strategies aimed at enhancing macrophage cholesterol export rather than suppressing macrophage populations.

Evidence that macrophage cholesterol burden can be modified in humans comes primarily from pulmonary alveolar proteinosis, where statin therapy has been associated with reduced AM cholesterol accumulation alongside improvements in radiological and physiological measures, supporting the feasibility of pharmacologically modulating macrophage sterol handling in vivo [67]. Translation of these observations to fibrotic lung disease remains uncertain. Large observational studies now report associations between statin use and reduced IPF risk and improved survival, but these analyses do not establish causality, disease-specific modification, or macrophage-centred mechanisms [68].

Importantly, macrophage-intrinsic responsiveness to cholesterol-modulating agents appears to be preserved. Ex vivo studies using primary human lung macrophages demonstrate that statin exposure can directly bias the macrophage transcriptional state toward a FABP4-aligned lipid-handling configuration, consistent with enhanced sterol processing and reduced lipid stress [69]. The alignment between these macrophage-intrinsic effects and favourable population-level associations supports sterol handling as a plausible therapeutic lever, while the absence of definitive disease modification highlights the limitations of systemic statin therapy and the need for macrophage-directed or lung-restricted approaches in IPF.

Together, these data position sterol efflux as a valid macrophage-centred therapeutic target while underscoring the importance of delivery, target engagement, and disease context. Future strategies will likely require approaches that are capable of enhancing cholesterol export and lipid handling within the alveolus without perturbing systemic lipid metabolism, particularly in the setting of established fibrosis.

### 5.3. Autotaxin–LPA Pathway Inhibition

Targeting the autotaxin–lysophosphatidic acid (LPA) pathway represents the most clinically advanced lipid-centred therapeutic strategy pursued to date in fibrotic lung disease. As outlined in Section 4, dysregulated LPA signalling integrates epithelial injury, macrophage activation, and fibroblast responses, providing a mechanistic rationale for therapeutic intervention.

Importantly, this lipid-signalling circuit is not confined to structural cells. Human bronchoalveolar lavage studies demonstrate that AMs are a significant source of autotaxin activity and are capable of generating bioactive LPA within the alveolar space, positioning macrophages as active contributors to local profibrotic signalling rather than passive responders [56]. These observations align with transcriptomic evidence that places disease-expanded macrophage states within lipid mediator and lysophospholipid-associated programmes in IPF.

Clinical translation of autotaxin pathway inhibition has progressed further than any other lipid-targeted approach in IPF. The autotaxin inhibitor GLPG1690 demonstrated favourable biomarker and lung-function signals in phase 2 studies but was discontinued during phase 3 development following safety concerns identified in interim analyses ([70] and reviewed in [71]). Earlier attempts to block downstream LPA signalling using first-generation LPA_1_ antagonists were similarly constrained by off-target toxicity, underscoring the challenges of systemically targeting broadly active lipid mediators.

More recently, next-generation LPA_1_ antagonists with improved pharmacokinetic and safety profiles have entered clinical evaluation. Preclinical development of BMS-986278 established potent and selective inhibition of LPA_1_ with antifibrotic activity in experimental models [71,72]. Building on this work, admilparant has completed the phase 2 evaluation in pulmonary fibrosis, demonstrating acceptable safety and exploratory efficacy signals that support continued investigation of LPA_1_ antagonism as a therapeutic strategy [73]. Although definitive disease modification has yet to be established, these studies collectively indicate that refinement of LPA pathway targeting, through improved selectivity, lung-restricted delivery, or combination approaches, remains a viable therapeutic direction.

### 5.4. Rebalancing Eicosanoid Networks

Altered eicosanoid signalling represents a potential therapeutic target in IPF. In the healthy alveolus, prostaglandin E_2_ (PGE_2_) produced by epithelial cells and AMs restrains fibroblast proliferation, myofibroblast differentiation, and extracellular matrix synthesis. In IPF, impaired induction of cyclooxygenase-2 is associated with reduced local PGE_2_ production, resulting in a loss of this antifibrotic restraint and increased fibroblast activation [28,29,52].

Studies further show that fibroblasts in fibrotic lung disease exhibit reduced responsiveness to PGE_2_ signalling, limiting the effectiveness of endogenous antifibrotic feedback even when PGE_2_ is present [29,52]. Together, reduced PGE_2_ availability and impaired downstream signalling diminish an important lipid-mediated control of matrix deposition in the alveolus.

In parallel, increased production of profibrotic eicosanoids promotes fibroblast proliferation and collagen synthesis. Prostaglandin F_2_α is elevated in IPF and correlates with disease severity and mortality, supporting an association with progressive fibrotic remodelling [54]. Experimental studies demonstrate that prostaglandin F_2_α directly stimulates fibroblast growth and extracellular matrix production.

Leukotrienes represent an additional macrophage-linked eicosanoid pathway relevant to fibrotic lung disease. In experimental models, macrophage-derived leukotrienes promote inflammatory cell recruitment and fibrotic remodelling, and inhibition of leukotriene synthesis attenuates fibrosis in vivo [53]. However, leukotriene-targeted therapies have not demonstrated consistent disease-modifying benefit in IPF [30].

Overall, these findings indicate that therapeutic modulation of eicosanoid pathways in IPF is unlikely to be achieved through single-mediator blockade alone. Approaches that restore antifibrotic lipid signalling while preserving broader macrophage lipid-handling capacity may be required to limit fibroblast activation and matrix accumulation in the alveolus.

### 5.5. Systemic Metabolic Modulators

Beyond lipid-specific pathways, several systemically acting metabolic agents have been explored for their capacity to modify profibrotic myeloid populations in fibrotic lung disease. These approaches do not target AMs directly, but provide insight into whether pathogenic myeloid states remain modifiable in established disease.

Clinical proof-of-mechanism that pharmacologic intervention can alter profibrotic myeloid populations in humans comes from a crossover pilot study of sirolimus, in which treatment was associated with a reduction in circulating fibrocytes in patients with IPF [74]. Although fibrocytes are distinct from AMs, they share overlapping transcriptional and functional features with disease-associated myeloid populations (reviewed in [75]), supporting the concept that profibrotic myeloid states are not irreversibly fixed.

Metformin provides a complementary example of a systemic metabolic agent with reported antifibrotic effects. In experimental models of lung fibrosis, metformin attenuates fibrotic remodelling through the suppression of NOX4-dependent signalling pathways [76]. Observational studies in patients with IPF and comorbid diabetes have suggested associations with reduced mortality or hospitalisation, although analyses across clinical trial cohorts have yielded mixed results [77,78]. Importantly, these studies do not resolve cell-type specificity and cannot distinguish direct effects on AMs from broader systemic actions.

Taken together, these findings indicate that profibrotic myeloid populations can be influenced pharmacologically in humans, but they do not yet define a macrophage-specific therapeutic strategy. In the context of IPF, such agents are best viewed as providing indirect support for the broader concept of myeloid state plasticity rather than as targeted interventions for AM reprogramming.

### 5.6. Targeting Epithelial Lipid Biosynthesis

An additional therapeutic opportunity lies in restoring epithelial PC remodelling, the reacylation step that maintains surfactant composition and alveolar lipid balance (via LPCAT1). Disruption of this process alters surfactant lipid composition, compromises epithelial stability, and creates an alveolar lipid environment that secondarily challenges macrophage lipid-handling capacity.

Recent experimental studies demonstrate that impaired PC remodelling contributes directly to fibrotic susceptibility by limiting AT2 progenitor function and perturbing intracellular lipid balance. Loss or reduction of LPCAT1 activity results in broad disturbances in phospholipid and cholesterol pools, providing a mechanistic link between epithelial lipid failure and downstream macrophage lipid stress [16,17]. These epithelial defects, therefore, act upstream of macrophage reprogramming, shaping the lipid flux to which resident macrophages must adapt.

Proof-of-concept studies indicate that epithelial PC remodelling can be pharmacologically enhanced in vivo, leading to improved epithelial repair and attenuation of experimental fibrosis [17]. Although such approaches primarily target epithelial metabolism, they are predicted to indirectly normalise macrophage lipid flux by stabilising surfactant composition and reducing exposure to oxidised or compositionally abnormal phospholipids within the alveolus.

Together, these findings position epithelial lipid biosynthesis as a complementary therapeutic target that may act in concert with macrophage-directed metabolic reprogramming. Rather than addressing macrophage dysfunction in isolation, restoring epithelial lipid homeostasis offers a means of upstream intervention that reduces metabolic stress across the alveolar unit.

### 5.7. Translational Considerations and Limitations

Despite strong mechanistic rationale, lipid-targeted therapies have thus far failed to demonstrate consistent disease-modifying efficacy in IPF. This likely reflects several fundamental biological constraints. IPF is spatially and temporally heterogeneous, such that macrophage states and lipid environments vary across regions and stages of disease, limiting the impact of uniformly delivered therapies. In addition, many lipid perturbations arise downstream of epithelial injury, meaning that targeting these pathways alone may not reverse the upstream processes sustaining fibrosis. Redundancy across lipid-signalling networks, including overlapping eicosanoid and lysophospholipid pathways, may further blunt the effect of single-pathway interventions. Finally, the macrophage metabolic state remains tightly coupled to epithelial-derived lipid flux, suggesting that effective therapy may require restoration of epithelial–macrophage interactions rather than modulation of macrophages in isolation. Together, these considerations indicate that lipid-centred strategies are likely to be context-dependent and stage-specific, and may be most effective when integrated with approaches that address epithelial dysfunction and tissue remodelling.

## 6. Conclusions and Future Directions

IPF can be understood as a disease in which disruption of alveolar lipid homeostasis intersects with epithelial injury, macrophage metabolic stress, and progressive fibroblast activation. Recurrent abnormalities in surfactant composition, impaired lipid synthesis by alveolar type II cells, and erosion of lipid-adapted macrophage homeostatic programmes consistently accompany the emergence of inflammatory and SPP1^hi^ macrophage states. While these associations are robust, much of the current evidence linking lipid abnormalities to macrophage state transitions remains correlative. In many cases, lipid perturbations may arise as downstream consequences of epithelial injury and altered surfactant handling rather than serving as primary drivers of macrophage reprogramming. Within this context, lipid abnormalities may nevertheless reinforce macrophage state transitions and contribute to the stabilisation of profibrotic microenvironments once epithelial homeostasis has been compromised.

A central implication of this framework is that the primary raison d’être of AMs is lipid metabolism. Unlike most tissue macrophages, AMs are specialised to reside within a lipid-rich airspace and to accommodate continuous surfactant uptake, processing, and disposal. Lipid handling is therefore not an auxiliary function but the dominant homeostatic task that defines AM identity. When epithelial lipid supply becomes excessive, compositionally abnormal, or oxidatively modified, this metabolic specialisation becomes a source of vulnerability, biasing macrophages toward stress-adapted and profibrotic states.

Viewing IPF through a lipid–macrophage lens shifts emphasis away from macrophage number or developmental origin and toward regulation of macrophage metabolic state. Disease-associated macrophage programmes appear to be sustained by persistent metabolic pressure within the alveolus, implying that these states are maintained rather than terminal. This distinction is important, as it suggests that macrophage dysfunction may remain modifiable without eliminating resident populations that are essential for alveolar maintenance and host defence.

This perspective also reframes therapeutic opportunity. Interventions that restore lipid balance, whether by improving epithelial lipid synthesis, enhancing macrophage lipid handling, or rebalancing bioactive lipid signalling, target upstream pressures that shape macrophage identity and epithelial resilience. Such strategies are conceptually complementary to existing antifibrotic therapies and may be most effective before fibrotic architecture becomes structurally entrenched.

Future progress will depend on resolving how specific lipid perturbations bias macrophage state transitions in human disease. Integrating lipidomic measurements with single-cell and spatial profiling will be essential for linking defined lipid alterations to macrophage programmes within their anatomical context. Functional studies using patient-derived epithelial cells and AMs will be required to identify points of reversibility, define temporal windows for intervention, and determine whether targeted modulation of lipid pathways can durably reset macrophage metabolic programmes in the distal lung.

Taken together, restoring balance within the alveolar lipid ecosystem provides a coherent explanation for disease persistence in IPF and a rational foundation for the development of next-generation, metabolism-informed therapies.

## Figures and Tables

**Table 1:** Functional enrichment evidence supporting lipid-metabolic modules. Functional enrichment evidence supporting the lipid-metabolic programmes defined in Table 2. Each module was supported by Gene Ontology Biological Process (GO:BP) enrichment with independent confirmation by KEGG pathway analysis. The GO:BP terms shown represent illustrative examples of enriched biological processes within each module, and the KEGG pathways indicate concordant metabolic or lipid-associated signalling themes. Enrichment analysis was used to validate the coherence of functional programmes rather than to infer pathway directionality or mechanistic causality.

Module	Supporting GO:Biological Process Terms (Examples)	KEGG Pathway Confirmation
AM2-1	Fatty-acid metabolic process; regulation of lipid storage; cholesterol storage	PPAR signalling; regulation of lipolysis
AM2-2	Glycerolipid metabolic process; lipid-metabolic process	Glycerolipid metabolism
AM2-3	Phospholipid metabolic process	Glycerophospholipid metabolism
AM2-4	Fatty-acid biosynthetic process	Biosynthesis of unsaturated fatty acids
AM2-5	Eicosanoid biosynthetic process; leukotriene metabolism	Arachidonic acid metabolism
AM2-6	Lipid transport; cholesterol efflux	ABC transporters
CD163-1	Cholesterol transport; intracellular sterol trafficking	Cholesterol metabolism
CD163-2	Lysosomal transport; sphingolipid catabolic process	Lysosome; sphingolipid metabolism
CD163-3	Lipoprotein metabolic process; cholesterol efflux	Fat digestion and absorption
CD163-4	Long-chain fatty-acid import; acyl-CoA metabolism	Fatty-acid metabolism
CD163-5	Phosphatidylcholine metabolic process	Glycerophospholipid metabolism
CD163-6	Response to oxidative stress-associated processes	Ferroptosis; xenobiotic metabolism

**Table 2:** Lipid-metabolic programmes defining alveolar macrophage states. Lipid-metabolic functional programmes organising AM states. Curated lipid- and metabolism-associated gene sets derived from Wendisch AM2 and CD163.LGMN macrophage populations were grouped into discrete functional modules aligned with FABP4-like and SPP1-like macrophage configurations. For each module, contributing lipid- and metabolism-associated genes identified by differential expression and functional curation are listed together with a concise biological interpretation reflecting dominant lipid-handling functions. Gene lists are illustrative rather than exhaustive and are intended to capture the defining features of each programme rather than to imply exclusivity or fixed lineage identity.

Module	Functional Programme	Contributing Lipid/Metabolic Genes	Biological Interpretation
AM2-1	Lipid droplet-associated lipid handling	*FABP4*, *PLIN2*, *HILPDA*, *PNPLA2*	Supports intracellular lipid buffering and accommodation of continuous surfactant-derived fatty-acid flux.
AM2-2	Glycerolipid metabolism and turnover	*LPL*, *AGPAT2*, *PNPLA2*	Enables dynamic triglyceride remodelling and lipid recycling under steady-state conditions.
AM2-3	Phospholipid metabolic processes	*GPD1*, *PNPLA6*, *AGPAT2*	Maintains phospholipid turnover and membrane lipid homeostasis in a lipid-rich environment.
AM2-4	Unsaturated fatty-acid biosynthesis	*SCD*, *ACOT7*	Supports the synthesis and modification of fatty acids required for membrane flexibility and lipid storage.
AM2-5	Eicosanoid and prostanoid metabolic capacity	*ALOX5*, *ALOX5AP*, *HPGD*	Confers capacity for regulated bioactive lipid mediator production without constitutive inflammatory signalling.
AM2-6	Lipid transport across membranes	*ABCG1*, *ABCG2*	Facilitates cholesterol and lipid efflux to prevent intracellular sterol accumulation.
CD163-1	Cholesterol transport and sterol homeostasis	*ABCA1*, *NPC1*, *NPC2*, *APOE*	Reorganises lipid handling toward sterol trafficking and stress-associated cholesterol management.
CD163-2	Endosome–lysosome lipid processing	*LAMP1*, *ASAH1*, *LIPA*	Supports lysosomal handling of lipid cargo under conditions of sustained lipid burden.
CD163-3	Lipoprotein particle handling	*APOE*, *PLTP*, *CD36*	Enables uptake and redistribution of extracellular lipoprotein-derived lipids.
CD163-4	Fatty-acid import and acyl-CoA metabolism	*CD36*, *ACSL3*, *ACSL4*	Biases lipid metabolism toward fatty-acid uptake and downstream oxidative or anabolic pathways.
CD163-5	Phospholipid metabolic processes	*LPGAT1*, *PLA2G7*	Remodel phosphatidylcholine species in response to altered lipid flux.
CD163-6	Oxidative/xenobiotic-linked metabolism	*HMOX1*, *CYP1B1*	Reflects engagement of oxidative stress-linked lipid metabolism under chronic injury conditions.

## Data Availability

No new data were created or analysed in this study.

## Abbreviations

The following abbreviations are used in this manuscript:

ABCA1	ATP-binding cassette transporter A1
ABCG1	ATP-binding cassette transporter G1
AGEs	advanced glycation end-products
AM	alveolar macrophage
AMPK	AMP-activated protein kinase
AT2	alveolar type II epithelial cell
COX-2	cyclooxygenase-2
DPPC	dipalmitoylphosphatidylcholine
FABP4	fatty acid-binding protein 4
GM-CSF	granulocyte–macrophage colony-stimulating factor
IPF	idiopathic pulmonary fibrosis
LPA	lysophosphatidic acid
LPC	lysophosphatidylcholine
LXR	liver X receptor
NOX4	NADPH oxidase 4
PAP	pulmonary alveolar proteinosis
PGE_2_	prostaglandin E_2_
PGF_2_α	prostaglandin F_2_α
PPARG	peroxisome proliferator-activated receptor-γ
ROS	reactive oxygen species
sRAGE	soluble receptor for advanced glycation end-products
SPP1	secreted phosphoprotein 1 (osteopontin)
